# Unfolding Resilience: Molecular Integration of the Integrated Stress Response and Mitochondrial UPR in Skeletal Muscle Homeostasis

**DOI:** 10.3390/muscles5020039

**Published:** 2026-05-22

**Authors:** Victoria C. Sanfrancesco, Daniella Della Mea, David A. Hood

**Affiliations:** Muscle Health Research Centre, School of Kinesiology and Health Science, York University, Toronto, ON M3J 1P3, Canada; vicsan3@my.yorku.ca (V.C.S.); dani2003@my.yorku.ca (D.D.M.)

**Keywords:** skeletal muscle, aging, muscle inactivity, mitochondria, stress response, exercise, ATF4, ATF5, CHOP, integrated stress response, unfolded protein response, adaptation

## Abstract

To maintain homeostatic conditions and optimal function during stressors, mitochondria initiate retrograde signaling. The mitochondrial integrated stress response (ISR) and unfolded protein response (UPR^mt^) are critical quality control mechanisms activated during instances of mitochondrial perturbations. Restoration of mitochondrial homeostasis is orchestrated by three transcription factors, ATF4, CHOP, and ATF5, which upregulate protective genes to counteract stress. As the health and function of skeletal muscle are heavily dependent on a highly adaptive mitochondrial network, defining how mitochondrial health is maintained across various conditions is essential. Although several studies demonstrate the importance of these responses following instances of stress, the signaling mechanisms required to initiate such pathways remain poorly characterized in skeletal muscle. This review examines how the mitochondrial ISR/UPR^mt^ and related transcription factors respond to organellar stress by emphasizing the molecular events that occur during exercise, aging and muscle disuse. By consolidating the literature, this work aims to highlight the current understanding of mitochondrial stress response signaling within skeletal muscle and thus emphasize areas for future research and potential therapeutic strategies during divergent metabolic conditions.

## 1. Introduction: Mitochondrial Function and Health

Mitochondria, colloquially termed the powerhouses of the cell, are responsible for producing the energetic molecule, adenosine triphosphate (ATP), from oxidative respiration (OXPHOS). These organelles possess their own double-stranded DNA, termed mitochondrial DNA (mtDNA), as a product of evolutionary endosymbiosis with α-proteobacteria [1]. Although the mitochondrial genome (mtDNA) only encodes 13 protein subunits of the mitochondrial electron transport chain (ETC), the remainder of mitochondrial proteins, ~1200, are encoded by the nuclear genome [2]. Therefore, the overall synthesis, maintenance, and structural capacity of mitochondrial function depend on the tightly coordinated regulation between both genomes [3,4].

While critical in cellular energy metabolism, mitochondria also play a pivotal role in regulating an array of biological functions, including calcium handling, redox homeostasis, programmed cell death (mitochondrially mediated apoptosis) and cellular inflammation [5]. Optimal health and function of these organelles is therefore required to sustain such diverse functions. With that being said, the overall quality of the mitochondrial network must be tightly regulated via coordinated communication between the mitochondria and nucleus (mito-nuclear) to integrate changes in the proteome [6,7]. Moderate levels of stress can trigger mito-nuclear communication, denoted as a retrograde stress response, to enhance organellar fitness and resilience against future insults [6,8,9]. This unique paradigm is typically denoted as “mitohormesis”, a non-traditional dose–response curve, in which manageable stress prompts beneficial mitochondrial adaptation [6,8,9]. Exposure to high-dose stressors, on the other hand, can produce maladaptive outcomes, triggering cell death and pathology [8,9,10]. Although mitohormesis is an established concept, the precise molecular signals that activate the mitochondrial stress response, as well as the mechanisms underlying its adaptive outcomes, remain poorly understood. In the current review, we discuss the mitochondrial stress response and its importance in maintaining organellar health, particularly within the context of skeletal muscle.

## 2. Skeletal Muscle and Mitochondrial Quality Control

Skeletal muscle is a central regulator of locomotion, whole-body energy expenditure, substrate utilization and thermogenesis [11]. In response to altered energetic demand or physiological stressors, such as exercise, skeletal muscle undergoes coordinated morphological, physiological and biochemical remodeling, reflecting its remarkable plasticity [12]. The adaptive capacity of skeletal muscle is thereby dependent on the highly efficient mitochondrial network, dictating the overall metabolic performance and health of skeletal muscle [5,13]. Given this, as alluded to previously, mitochondria must therefore preserve proteostatic, bioenergetic, and redox homeostasis through tightly regulated mitochondrial quality control (MQC) mechanisms [14]. These processes are identified as mitochondrial biogenesis, which drives the synthesis of new organelles; mitophagy, responsible for selectively removing dysfunctional mitochondria; and the transient activation of a mitochondrial stress response [15,16]. Collectively, such quality control mechanisms safeguard mitochondrial network integrity in order to meet and sustain the energetic demands of skeletal muscle [15,16].

Unlike biogenesis and mitophagy, the mitochondrial stress response dynamically senses perturbations in the mitochondrial network to coordinate adaptive transcriptional programs. This response aims to restore organellar integrity, positioning this mitohormetic regulatory process as a central mediator of mitochondrial network resilience and plasticity [6]. The mitochondrial stress response is a coordinated event that can be categorized into the initial general phase of detection, termed the integrated stress response (ISR), which ultimately dictates the fine-tuning of a more centralized stress program, the mitochondrial unfolded protein response (UPR^mt^) [6,17,18,19]. Multiple mitochondrial insults can trigger the activation of the ISR/UPR^mt^, eliciting transcriptional and translational reprogramming [6,17,18,19]. The overall nuclear response of the ISR/UPR^mt^ is mediated by three critical transcription factors, ATF4, CHOP, and ATF5, which upregulate the expression of protective genes to combat the associated mitochondrial stressor [19]. While evidence has suggested a newfound role for the stress response and associated regulators in maintaining mitochondrial health, less is known about how this restorative mitohormetic program operates in skeletal muscle during instances of physiological stress, such as during exercise, aging and chronic muscle disuse.

It is well established that global MQC activation in skeletal muscle is context-dependent, occurring in response to metabolically divergent stimuli, including exercise-induced metabolic stress and prolonged periods of muscle inactivity [20]. In the former, the stimulation of the MQC program with contractile activity promotes coordinated adaptation of mitochondrial and muscle phenotypes; however, with muscle inactivity, such processes are denoted as maladaptive and thus incapable of rescuing cellular homeostasis [14,21]. Although studies investigating MQC signaling in skeletal muscle during exercise training and muscle inactivity (including aging and muscle disuse) have largely focused on alterations in mitophagy and mitochondrial biogenesis, the overall contribution of mitochondrial stress response signaling (ISR and UPR^mt^) and associated transcriptional regulators remains comparatively underexplored. Accordingly, this review synthesizes current evidence defining the cascades that govern mitochondrial-specific activation of the ISR and UPR^mt^, with an emphasis on skeletal muscle adaptation/maladaptation (either following exercise or muscle inactivity). By framing the mitochondrial stress response as a continuum rather than discrete pathways, we highlight its central role in skeletal muscle plasticity and health, identify critical gaps in the literature, and propose key areas for future investigation.

## 3. A Brief History of Mitochondrial Stress Response

The concept of a hormetic mitochondrial stress response was first established by pioneering work from Vandana Parikh and colleagues in 1987 [22], demonstrating that mitochondrial dysfunction, particularly mutations or depletion of mtDNA, alters nuclear gene expression in yeast. Following this work, subsequent studies revealed that mitochondria possess robust intermediary mechanisms to preserve cellular function and enhance their adaptive capabilities under various stressors [6]. In the face of acute organelle stress, mitochondria can elicit a coordinated stress response through the generation of a mito-nuclear retrograde signal, as mentioned previously. This consequently initiates the enhancement of protective nuclear genes encoding mitochondrial proteins (NuGEMPs) [6,16,17]. Such transcriptional reprogramming serves to resolve the original organellar perturbations and promote the improvement of mitochondrial function and adaptation [6,16,17,23]. Building on these initial findings, later investigations established the ISR/UPR^mt^ in mammalian cells. In 1996, Martinus and colleagues showed that mtDNA loss ($\rho^{0}$) via Ethidium Bromide treatment in rat hepatoma cells (H4) led to increases in ISR/UPR^mt^ downstream genes, mitochondrial chaperones, via elevations in their transcriptional activity [24]. This work was one of the first to emphasize, in mammalian cells, that mitochondrial defects trigger a retrograde response, which is now classified as the ISR/UPR^mt^. One step forward, Zhao and colleagues, in 2002, determined that CHOP, an ISR/UPR^mt^ transcription factor, is required for the induction of mitochondrial stress-responsive proteins in COS-7 cells following mitochondrial protein toxicity [25]. Subsequently, further experimental analyses identified both ATF4 and CHOP, ISR/UPR^mt^ transcription factors, as dual regulators of the retrograde signaling response in lymphocytes subjected to rotenone-induced inhibition of Complex I in the ETC [26,27]. Collectively, these studies defined a previously unrecognized, mitochondrial-specific stress response, largely orchestrated by ISR/UPR^mt^ transcription factors. These advances catalyzed investigations into the role of this stress response program in preserving mitochondrial health across yeast, nematodes, mammalian cells and mouse models. Appreciating the physiological importance of the ISR/UPR^mt^, therefore, requires a precise mechanistic understanding of how this pathway is initiated, integrated, and ultimately resolved at the molecular level, a complexity that the subsequent sections of this review aim to address.

## 4. The Integrated Stress Response (ISR): A Conserved Cellular Sentinel

### 4.1. General Activation and Regulation of the ISR

The ISR is an evolutionarily conserved adaptive program that is responsive to a variety of different global environmental and pathological stressors [18]. Such stressors include protein homeostasis (proteostasis) defects, nutrient deprivation, viral infection, oxidative stress, and (most recently) mitochondrial dysfunction/perturbations [27]. The ISR serves to maintain cellular health and overcome stressors by reprogramming gene expression via the activation of specific transcription factors: ATF4, CHOP and ATF5 [28,29]. In terms of overall activation, this program becomes responsive to distinct stress signals that are initially sensed and transmitted through the induction of four serine/threonine upstream kinases, protein kinase RNA-like endoplasmic reticulum kinase (PERK), general control nonderepressible 2 (GCN2), RNA-activated protein kinases (PKR), and heme-regulated inhibitor kinase (HRI) [30]. These four kinases share extensive homology in their catalytic domains but possess distinct regulatory domains, reflective of their unique response to stressors [30]. Upon sensing stress, the kinases will undergo full activation via autophosphorylation and homodimerization events, where they will then converge on a common target, the eukaryotic translation initiation factor eIF2, a heterotrimer consisting of α, β, and γ subunits [31]. Specific to the ISR, the α subunit is phosphorylated at Serine 51 by one or more of the four kinases, leading to reduced global translation as well as selective synthesis of important regulators of homeostatic control [32,33,34]. This distinct regulation ultimately alleviates the pressure of continued translation, which can be damaging to cellular constituents that are already facing stress, by primarily focusing on restoring cellular homeostasis and thus overall survival.

The prominent feature of ISR signaling lies in its alterations of the ternary complex (TC), which is instrumental in initiating the translation of cap-dependent translation, commonly identified as AUG-initiated upstream open reading frames (uORFs) in the cell’s transcriptome [35]. The TC is composed of the heterotrimeric eukaryotic translation initiation factor eIF2, bound with guanosine 5′-triphosphate (GTP), and a charged methionyl-initiator tRNA (Met-tRNA_i_) [27]. Under basal conditions, the TC will dock onto the 40S ribosomal subunit, creating the 43S preinitiation complex (PIC) [36]. The recognition of the start codon AUG on mRNA triggers GTP hydrolysis of eIF2 and its release, followed by the recruitment and binding of the 60S ribosomal subunit, thus allowing the elongation phase of protein synthesis to commence [36]. The GDP bound to eIF2 is then exchanged for GTP, recycling eIF2 to its active state, allowing for another round of translation initiation [27]. This recycling is catalyzed by the interaction with the guanine exchange factor (GEF) protein, eIF2B [37]. Briefly, eIF2B is composed of two copies of five different subunits (*α*, *β*, *δ*, *γ*, and *ε*) that assemble into a large two-fold symmetric heterodecamer; however, eIF2B’s catalytic nucleotide exchange activity resides in the eIF2B*ε* subunit [38]. In order to exchange GDP for GTP, eIF2B must form a complex with its substrate, eIF2. However, phosphorylation of eIF2α induces a profound rearrangement in this subunit’s structure, which alters its binding site on eIF2B, thereby restricting the nucleotide-binding domain and preventing access to the catalytic GEF domain [37,38]. This inhibition subsequently blocks the eIF2B-mediated exchange of GDP for GTP and reduces the formation and availability of the 43S PIC, rendering the cell unable to re-initiate protein translation and thus attenuating global translation of 5′ cap-dependent mRNAs [36,37,38]. Simultaneously, the translation of mRNAs that contain short “inhibitory” upstream open reading frames (uORFs) in their 5′ untranslated region (5′UTR) is enhanced [39]. Under basal/normal conditions, these “inhibitory” uORFs prevent the translation of downstream coding DNA sequences (CDSs) by causing the translation machinery to “skip” the start codon of the sequence; however, the reduced availability of the TC when eIF2α is phosphorylated allows the inhibitory uORF to be skipped, enabling the downstream coding sequence to be expressed [27]. As a result of this, the expression of selective transcripts that contain short “inhibitory” upstream open reading frames (uORFs) in their 5′ untranslated regions will be elevated [28]. The most notable mRNAs translated constitute the ISR transcription factors, ATF4, CHOP, and ATF5, which, in concert, mediate the nuclear expression of protective genes [40,41,42,43,44].

The ISR is terminated by the dephosphorylation of the central signaling molecule, eIF2α. This event typically occurs when the initial stressor has been relieved, warranting normal protein synthesis and cell functioning to be resumed. The dephosphorylation of eIF2α is mediated by a protein phosphatase 1 (PP1) complex that recruits a catalytic subunit (PP1c) and a regulatory subunit [45]. In mammals, the most prevalent regulatory subunits of the phosphatase activity are GADD34, known as growth arrest and DNA damage-inducible protein, and CReP, the constitutive repressor of eIF2α phosphorylation [46,47]. In a complex with PP1c, CReP, in unstressed conditions, is ubiquitously responsible for monitoring the basal levels of eIF2α phosphorylation [47]. Conversely, GADD34, which is a downstream target of the ISR, specifically during the later stages of response activation, forms a complex with PP1c to significantly increase eIF2α dephosphorylation, acting as an important negative feedback loop to restore protein synthesis [46].

### 4.2. Mitochondrial Stress-Specific Activation of the ISR

**Figure 1:**
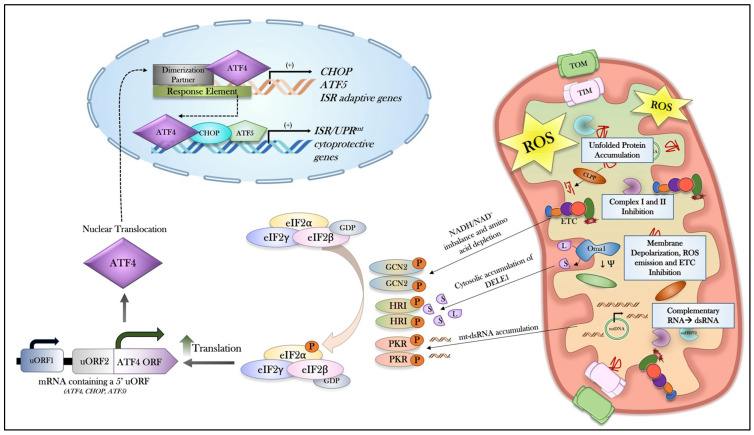
The activation of the ISR during mitochondrial stress. Various mitochondrial stressors, such as unfolded protein accumulation, ETC inhibition, membrane depolarization and ROS emission, can initiate the ISR. Such stressors can trigger various physiological changes, ranging from metabolic alterations to the accumulation of DELE1 and mt-dsRNA within the cytosol, activating the ISR kinases GCN2, HRI, and PKR. Once activated, the ISR kinases will converge on the α-subunit of the eIF2 complex and induce its phosphorylation, initiating the selective translation of mRNA containing an inhibitory upstream open reading frame (uORF), namely, ATF4. Once ATF4 is translated, it will translocate to the nucleus, where it can form heterodimers to induce the expression of CHOP, ATF5 and ISR adaptive genes to combat the initial mitochondrial stressor and restore mitochondrial function/homeostasis. In concert, ATF4, CHOP and ATF5 can further propagate the adaptive response of the ISR.

The ISR has emerged as a fundamental adaptive pathway in which cells can sense and respond to mitochondrial dysfunction by coordinating a broad mitohormetic transcriptional program, ultimately to preserve organellar homeostasis. A diverse array of mitochondrial insults, including pathological increases in ROS emission, defects in DNA replication/mtRNA processing, ETC blockage/inhibition, protein toxicity, and reductions in mitochondrial membrane potential, can induce the ISR (Figure 1) [43,48,49,50,51,52]. The breadth of stimuli capable of engaging the ISR underscores its role as a central surveillance program and an indispensable guardian of mitochondrial integrity. Despite this growing appreciation, the molecular mechanisms by which mitochondrial stress signals are sensed and transduced to the ISR machinery remain poorly understood. Recent interest has converged on the upstream regulatory kinases, HRI, GCN2, and PKR, as critical mediators capable of detecting mitochondrial perturbations and initiating the ISR program. However, the specific conditions governing their selective activation, the degree to which they operate redundantly or cooperatively, and how their outputs are integrated at the level of shared downstream effectors remain active areas of investigation. This section solely examines the mechanisms by which each ISR kinase can become activated in response to mitochondrial stressors.

#### 4.2.1. HRI and Mitochondrial Stress

The most prominent and well-explored kinase responsible for the initiation of the ISR following mitochondrial stress is HRI. Multiple lines of evidence have indeed demonstrated that the global activation of HRI by mitochondrial stressors can impart restoration of organellar function [50,53,54,55,56,57]. For example, under chemical stress or genetic conditions that induce mitochondrial perturbations, pharmacological activation of HRI can markedly restore mitochondrial function and morphology [53]. However, the signaling axis underlying HRI activation in response to mitochondrial stress was only recently uncovered by two independent research groups, revealing a pathway involving the mitochondrial stress-responsive metalloprotease OMA1 [58] and the mitochondrial protein DELE1 [59,60]. Guo and colleagues first identified that the presence of various mitochondrial stressors, such as oligomycin, antimycin A, rotenone, and CCCP, can trigger the ISR in various cell lines, including HEK293T, HeLa, and iPSC cells [60]. Parallel to these findings, Fessler and colleagues further reinforced the OMA1-DELE1-HRI pathway by treating a multitude of cell lines, including HAP1 and SH-SH5Y cells, with mitochondrial inhibitors [60]. Consistently, both studies elucidated that OMA1, ordinarily dormant under physiological conditions, is rapidly activated by mitochondrial stressors, such as ETC dysfunction and mitochondrial membrane depolarization [61,62], to engage with the imported longform DELE1 (L-DELE1) and proteolytically cleave it at the N-terminus into a shorter peptide fragment (S-DELE1) [63]. Some evidence also suggests that S-DELE1 will stall in the TOM complex, triggering the release and ejection of the fragment into the cytosol to accumulate, leading to the interaction with HRI [59,60,64]. The N-terminal tetranucleotide repeats present in DELE1 facilitate protein–protein interactions and, in this case, are capable of binding to and influencing the autophosphorylation status of HRI [59]. In addition, more recent findings have also identified that in the face of mitochondrial import stress and iron deficiency, L-DELE1 can accumulate in the cytosol to initiate an OMA1-independent activation of HRI in HeLa and HEK293T cells [65]. These pivotal studies highlight the foundational connection between mitochondrial stress and ISR activation via the OMA1-DELE1-HRI pathway.

#### 4.2.2. GCN2 and Mitochondrial Stress

Mounting evidence has demonstrated that GCN2 is also required to maintain mitochondrial quality during stress, particularly in response to elevations in ROS, ETC dysfunction, and the imbalance of Krebs Cycle derivatives and associated amino acids [66,67,68,69,70,71]. Although seemingly critical in the preservation of mitochondrial homeostasis, the mechanisms underlying GCN2 activation, and thus, context specificity, are poorly understood within the literature. For example, two research groups consistently revealed that mitochondrial ROS increase the overall level of GCN2 phosphorylation [69,70]. However, the molecular underpinnings of this activation remain elusive. In a different context, evidence in C2C12 myoblasts and myotubes has established a potential signaling paradigm for GCN2 during mitochondrial stress, particularly during ETC dysfunction [29,43]. In the aforementioned study, Mick and colleagues demonstrated that Complex I and II inhibition led to a heightened ratio of NADH/NAD^+^, which restricts aspartate production through the Krebs Cycle halting of oxaloacetate (OAA) transamination, leading to the depletion of its derivative asparagine and, thus, amino acid deprivation [29,72,73,74]. This decline will activate GCN2, which then triggers the induction of the ISR, most likely driven by reductions in charged tRNA.

#### 4.2.3. PKR and Mitochondrial Stress

Lastly, PKR has also been associated with responding to mitochondrial perturbations, such as unfolded proteins, alongside cytosolic mtDNA [75]. PKR is classically known to recognize double-stressed RNA (dsRNA) primarily through viral interactions; however, this kinase can also sense dsRNA molecules derived from mtRNA and, thus, mtDNA [75]. Since the mitochondrial genome is circular and double-stranded, bidirectional transcription from mitochondrial DNA light (L) and heavy (H) strands leads to the transcription of precursor complementary RNA molecules, which can hybridize to form mitochondrial dsRNA molecules [76,77,78,79]. mtRNA can be degraded by the degradosome complex, which consists of the mitochondrial helicase SUV3 and polynucleotide phosphorylase (PNPase) [76,77,78,79]. However, disruptions in mtRNA degradation processes, which typically occur during mitochondrial stress and proteostasis failure, can lead to the accumulation of complementary RNA molecules, thereby increasing the likelihood of dsRNA formation [76,77,78,79]. The association between PKR and mtRNA was first eloquently elucidated in a study by Kim and colleagues in 2018. Using formaldehyde-mediated crosslinking and immunoprecipitation sequencing (fCLIP-seq), HeLa cells treated with the apoptotic inducer Staurosporine experienced an increase in mtRNA production and, thus, mtRNA-PKR interaction [80]. Following these findings, the activation of PKR during various mitochondrial stressors was thoroughly examined in a multitude of cell lines [55,81,82,83,84]. For example, in the most recent study by Kusuma and colleagues, treatment using a mitochondrial chaperone inhibitor, gamitrinib–triphenylphosphonium (GTPP), to impair mitochondrial protein handling (proteostasis) in MEFs led to the formation and subsequent release of mt-dsRNA into the cytosol via BAX/BAK channels, conferring cytosolic sensing and activation of PKR to initiate the ISR [81].

While the aforementioned studies have demonstrated that the ISR kinases can respond to mitochondrial perturbations, the precise context of their activation, particularly within skeletal muscle, remains to be fully elucidated.

#### 4.2.4. Kinase-Independent Activation of ISR

Beyond canonical ISR kinase signaling, mitochondrial stress can also initiate the ISR via mTORC1-mediated post-translational modifications. In cultured MEFs, mTORC1 activation drives ATF4 accumulation and downstream gene expression even in cells harboring a Serine51–Alanine knock-in mutation of eIF2*α*, in which eIF2*α* phosphorylation is abolished [85]. More recent evidence in HEK293T cells further demonstrated that ATF4 is a target substrate of mTORC1 [86,87]. During Doxorubicin-induced mitochondrial stress, mTORC1 can phosphorylate ATF4 at two specific residues, Serine 166 and Threonine 173 [86]. These post-translational modifications, which occur independently of eIF2*α* activation, are sufficient to initiate an ATF4-dependent ISR program and thereby preserve mitochondrial homeostasis during organellar insults [86]. Consistent with these findings, mutation of these phosphorylation sites markedly attenuates ATF4-mediated ISR induction, diminishing the expression of known gene targets, including *Hspa9* and *Asns* [86]. Although this non-canonical pathway refines our understanding of ISR regulation, it remains unclear whether similar mTORC1-ATF4 signaling operates in skeletal muscle to support muscle integrity during mitochondrial stress.

### 4.3. Downstream Consequences of ISR Activation: Mitochondrial Stress Resolution

Following the activation of the ISR, translation is preferentially directed toward a subset of stress-responsive mRNAs, notably those encoding the ISR transcription factors ATF4, CHOP, and ATF5, as described above. Although all three factors contribute to the coordination of the ISR in mammalian cells, seminal work by Quirós and colleagues in 2017 first established ATF4 as the principal effector of this program, acting upstream of CHOP and ATF5 [52]. This was evaluated through comprehensive multi-omics in HeLa cells, in which mitochondrial stress was induced via pharmacological inhibition of mitochondrial import, OXPHOS functioning, ribosomal translation, and membrane potential [52]. From this work, alongside various other studies, it was subsequently revealed that ATF4 drives a distinct mitohormetic transcriptional program that promotes the expression of genes involved in metabolic reprogramming, (serine biosynthesis, one-carbon metabolism, amino acid synthesis and transport), protein quality control (chaperones and proteases), protection against oxidative damage (glutathione synthesis, cysteine availability, and NADH production), and autophagy/mitophagy enhancement to remove damaged organelles [50,88,89,90,91,92,93,94,95,96,97,98,99,100].

This diverse transcriptional control reflects the ability of ATF4 to dimerize with a wide variety of bZIP proteins (heterodimers), which includes three major subfamilies: c-Jun/c-Fos, ATF/CREB and CCAAT enhancer binding protein (C/EBP). In such heterodimers, ATF4 can bind to a multitude of promoter response elements (REs), including amino acid (AARE), C/EBP-ATF (CARE), cAMP (CRE), antioxidant (ARE), and mitochondrial (MURE) response elements via the flexible recognition of the DNA-motif 5′-GTGACGT[AC][AG]-3′ [101,102,103]. These interactions have been characterized across multiple cell types through chromatin immunoprecipitation and sequencing (ChIP-seq) [103]. An exhaustive catalog of more than 200 putative ATF4 downstream targets, along with the functional heterodimerization factors/partners (~40 binding partners), such as CHOP and ATF5, was recently compiled by Neill and Masson in 2023, thereby illustrating the complexity of ATF4 regulation [104].

Building on these foundational studies, more recent findings have delineated a temporal activation of the ISR within multiple tissue types, including skeletal muscle, which is characterized by discrete phases and transcriptional outcomes, summarized below [50]. Early-phase activation of the ISR is marked by the preferential induction of stress-associated myokines, including FGF21 and GDF15, together with transcriptional regulators linked to metabolic rewiring, such as CHOP and ATF5, with global translation simultaneously attenuated. This stage aims to conserve cellular energy while providing a temporary alleviation and preparation to resolve metabolic stress. The second phase encompasses the bulk of metabolic reprogramming, marked by coordinated upregulation of gene networks that drive de novo serine biosynthesis (*Psat1*, *Psph*, *Phgdh*), one-carbon metabolism (*Mthfd2*, *Shmt2*), glutathione production (*Slc7a11*, *Slc3a2*), and amino acid synthesis (*Asns*, *Cth*). This stage induces a metabolic shift to redirect carbon flux from glycolysis to support the aforementioned processes. The final phase transitions towards the activation of the mitochondrial unfolded protein response (UPR^mt^), culminating in an increase in the expression of genes that support protein folding and handling (*Hspa9*, *Hspd1*, and *Hspe1*), mitochondrial proteostasis (*Lonp1*), adaptive mitophagy/autophagy signaling (*Lc3b*, *Atg7*, *Prkn*) and antioxidant defense (*Hmox1*, *Nfe2l2*) [50].

Although the ISR is a mitohormetic mechanism that promotes cellular adaptations through transient metabolic reprogramming and compensatory pathways, the precise transcriptional signature elicited by mitochondrial stress remains incompletely defined. In particular, it is unclear whether this conserved, phase-dependent ISR gene program is uniformly engaged in skeletal muscle during physiologically relevant mitochondrial stressors, such as exercise or throughout aging. Confirming this fact in skeletal muscle would provide a better understanding of how the mitochondrial stress response operates and is functionally important in the maintenance of mitochondrial health.

### 4.4. Cell Guardian or Executioner? The ISR as a Double-Edged Sword

While the activation of the ISR is generally regarded as protective during moderate stress, under conditions when the stressor is severe, prolonged, or exceeds the capacity to restore homeostasis, the ISR can promote cell death via the regulation of various pro-apoptotic effectors [105]. This dual-phase switch is largely dependent on the duration, intensity, and context of the stressor, shifting its mitohormetic balance towards maladaptation. However, the precise molecular trigger that dictates this outcome remains unknown. During prolonged stress, ATF4, upon hyper- or sustained-activation, can regulate a gene expression program that initiates cell death predominantly through CHOP activation [27,106,107]. With persistent elevation in stressed cells, CHOP can transform its role from a protective modulator to a driver of apoptosis by upregulating the expression of pro-apoptotic BH3-only proteins—BIM, PUMA, NOXA, BID, transmembrane death receptors, 4 (DR4) and 5 (DR5)—while concurrently repressing anti-apoptotic Bcl-2 proteins like MCL1, BCL-XL, and BCL2 [54,107,108,109,110,111,112,113,114]. This CHOP-mediated cell death program can trigger the intrinsic mitochondrial-mediated apoptotic pathway through the preferential binding of pro-apoptotic BH3-proteins to anti-apoptotic proteins at the BH3-binding groove. This consequently displaces the inhibitory binding on the pro-apoptotic effector, BAX, which acts on BAK at the mitochondrial outer membrane (MOM) [115,116]. Since BAK is constitutively embedded in the MOM, BAX, upon activation and translocation from the cytosol, will oligomerize at the MOM to displace the inhibitory VDAC2 binding on BAK to thereby initiate BAK oligomerization [117]. These oligomerization events, in turn, facilitate mitochondrial outer membrane pore permeabilization (MOMP), permitting the induction of mitochondrial-mediated apoptotic signaling through the release of various pro-apoptotic factors, such as cytochrome c, apoptosis-inducing factor (AIF), and Endo G [118,119]. BAX and BAK can also be activated more directly, mediated by the cleavage of pro-apoptotic BH3-proteins, such as BID and BIM [120,121]. Indeed, multiple studies have confirmed this association of CHOP and apoptosis in various cellular stress conditions. For example, Puthalakath and colleagues observed an induction of CHOP and the expression of BIM in several cell types upon endoplasmic reticulum stress [122]. qPCR analyses confirmed that CHOP, when dimerized with C/EBPɑ, binds to a regulatory site on the BIM promoter, increasing its transcription during cellular stress and promoting apoptosis. In the same study, with tunicamycin treatment, an endoplasmic reticulum (ER) stress inducer, CHOP knockout thymocytes had a greater survival rate compared to WT thymocytes, supporting that this pathway is critical for stress-induced apoptosis. In cortical neuronal cells transfected with an siRNA directed against CHOP, PUMA mRNA was reduced after tunicamycin induction, leading to an attenuation in apoptosis [123]. This highlights the regulatory effects of CHOP on stress-induced apoptosis through the control of BH3-only proteins.

Although the ATF4-CHOP-BAX/BAK axis represents the canonical route by which ISR activation converges on an apoptotic commitment, it is now appreciated that the ISR can also drive programmed cell death through several mechanistically distinct pathways. One such pathway involves a non-canonical, cell-autonomous role of the CHOP target, transmembrane death receptor 5 (DR5), which functions as a key mediator of ISR-induced apoptosis, independently of its classical activation by extracellular ligands (such as TNF-related apoptosis-inducing ligand (TRAIL)) [124,125]. Persistent ISR signaling via the ATF4-CHOP axis directly upregulates DR5 expression, and upon surpassing a critical threshold, DR5 undergoes oligomerization and forms a death-inducing signaling complex (DISC), activating Caspase-8 to trigger mitochondrial apoptosis through BID cleavage [126,127,128]. This ligand-independent DR5 activation has been supported by genetic and pharmacological studies, including the use of an ISR inhibitor, such as ISRIB, which attenuates DR5 induction and cell death, confirming the centrality of the eIF2α-ATF4-CHOP-DR5 axis in this apoptotic switch [124,129,130]. Notably, the functional significance of DR5 in ISR-mediated apoptosis is further underscored by evidence that genetic ablation of DR5 abrogates cell death in response to prolonged stress, positioning DR5 as a general kill switch for the terminal ISR [124]. Nevertheless, the precise molecular cues that govern DR5 oligomerization and activation in the absence of ligands, as well as the interplay between mitochondrial and death receptor pathways under diverse stress conditions, remain active areas of investigation. Collectively, these findings delineate a model in which the ISR, through the eIF2*α*-ATF4-CHOP axis, integrates stress signals to determine cell fate, with DR5 serving as a critical effector of apoptosis when adaptive capacity is exceeded.

While this death-associated pathway has been explored in multiple cell lines, the extent to which these signaling cascades occur in skeletal muscle to potentially induce atrophy is still unknown. Future work is therefore required to mechanistically elucidate this signaling paradigm and its relationship to skeletal muscle health during atrophy-inducing conditions.

### 4.5. The UPR^mt^ in Mammalian Cells

**Figure 2:**
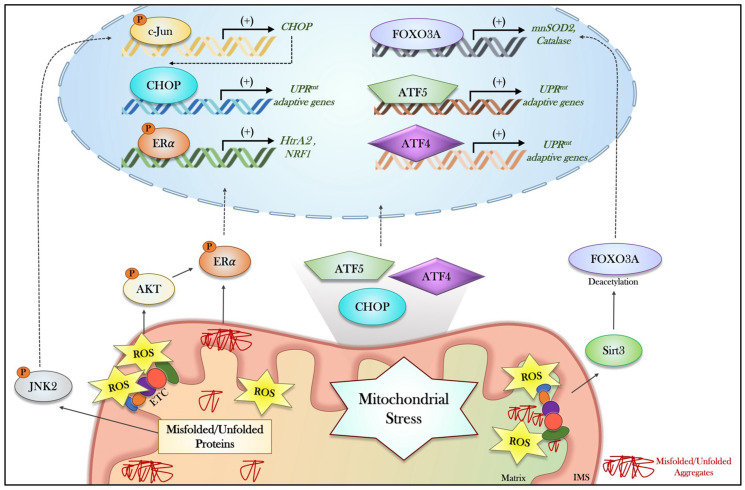
The induction of the mitochondrial unfolded protein response (UPR^mt^). The canonical UPR^mt^ axis, involving ATF4, CHOP and ATF5, is activated following global mitochondrial stress, such as misfolded/unfolded protein accumulation, elevations in ROS emission and imbalanced protein status, to induce the expression of related protective genes, such as chaperones and proteases. Other non-canonical pathways of the UPR^mt^ can be initiated during instances of specific compartmental stressors. In the intermembrane space (IMS), ROS can trigger the activation of the kinase AKT to then phosphorylate ERα, where it will localize to the nucleus to upregulate the expression of NRF1. Similarly, misfolded/unfolded protein aggregates within the IMS can also directly prompt the activation of ERα to stimulate the expression of HtrA2. In the matrix, elevations in both ROS and misfolded protein aggregates can elicit Sirt3 deacetylase activity, which will then deacylate FOXO3A, initiating its nuclear activation and regulation of mnSOD2 and catalase. These signaling axes all contribute to the remediation of mitochondrial function during various perturbations. Solid arrows indication an activation event, dashed arrows indicate nuclear movement.

As previously discussed, activation of the ISR orchestrates a phase-specific initiation of the UPR^mt^ within mammalian cells, thereby serving as an essential precursor to UPR^mt^ induction primarily through the upregulation of ATF4, CHOP and ATF5. Although these factors are critical for the UPR^mt^, their coordination in mitochondrial health has only recently been partially defined [50,93]. Moreover, while the UPR^mt^ and associated mitohormetic mechanisms have been clearly characterized in the model organism, *C. elegans* [131,132], in mammalian cells, the precise signaling governing this program remains elusive. Therefore, this section highlights the canonical signaling mechanisms underlying the activation of the mammalian UPR^mt^, with a focus on the three aforementioned regulators, alongside alternative modes of non-canonical UPR^mt^ activation (Figure 2).

The UPR^mt^, like the ISR, is an adaptive transcriptional program that preserves mitochondrial homeostasis during cellular stress [133]. This mitohormetic program has evolved over time to maintain optimal mitochondrial functioning by regulating the folding and quality control of the mitochondrial proteome, especially since mitochondria greatly rely on the import of nuclear-derived mitochondrial proteins for the assembly of ETC complexes [134,135]. In mammalian cells, it was first demonstrated that the presence of unfolded/misfolded proteins and mtDNA depletion enhances the expression of the stress-responsive chaperones HSP60/10 and proteases ClpP and mtDnaJ [24,25], a response thought to be solely mediated by CHOP [25,136]. During mitochondrial stress, CHOP induction depends on the activation of the kinase JNK2, which phosphorylates and activates the transcription factor c-Jun, leading to the preferential elevation in CHOP expression [25,136]. Once induced, CHOP binds to specific promoter elements within UPR^mt^-related genes to thereby upregulate their expression and initiate a restorative response [137]. However, later findings indicated the presence of additional conserved response elements within the promoter region of UPR^mt^ targets, suggesting that CHOP alone is insufficient to fully elicit this response [137].

Subsequent work identified ATF5 as the second core factor of the UPR^mt^ and the mammalian homolog of ATFS-1, the central regulator of the UPR^mt^ in *C. elegans* [138]. This functional conservation was supported by the restoration of mitochondrial function in ATFS-1-deficient *C. elegans* upon the exogenous expression of ATF5 [138]. It has since been acknowledged that ATF5, like ATFS-1, contains both a bZIP domain and a mitochondrial targeting sequence, allowing for both its nuclear and mitochondrial localization, dictated by the presence of mitochondrial stress [98,134,138]. Under basal conditions, ATF5 will be imported into the mitochondria and subsequently degraded by resident proteases (LONP1); however, upon mitochondrial stress, such as proteotoxicity and elevations in ROS, import capacity will be blunted, and ATF5 will instead localize to the nucleus to transcriptionally regulate stress-responsive genes [138]. Beyond ATF5, UPR^mt^ signaling is considerably divergent between *C. elegans* and mammalian cells, including the main transcriptional regulators and additional signaling mechanisms.

While CHOP and ATF5 were clearly elucidated as central factors in canonical UPR^mt^ signaling in earlier work, the contribution of ATF4 has only recently been defined. As discussed previously, work in 2017 was the first to elucidate the role of ATF4 in the UPR^mt^, as global transcriptomics revealed the presence of ATF4-binding elements in the promoters of stress-responsive genes [52]. In addition, following various mitochondrial stressors, de novo motif analysis of common upregulated genes was conducted and revealed a similar binding element motif recognized by both ATF4 and CHOP [85]. However, ATF4 retained the highest statistical association with this binding element, positioning ATF4 as the central regulator of the mitochondrial stress response and thus the UPR^mt^. Moreover, in the same study, HeLa cells lacking a functional copy of ATF4 failed to upregulate several UPR^mt^-related genes while exhibiting an inability to restore mitochondrial functioning [52]. This work was therefore one of the first to connect ATF4 as an additional regulatory factor of the UPR^mt^, alongside CHOP and ATF5, with a potentially greater role for ATF4 than once hypothesized [52]. This association was further proven in alveolar epithelial cells, implicating ATF4 as a critical rheostat in the UPR^mt^ and positioning itself beyond CHOP and ATF5 [139]. During oligomycin-induced mitochondrial stress, the absence of ATF4 but not ATF5 reduces the activation of the UPR^mt^, as evidenced by reductions in the expression of related genes, such as chaperones and proteases [139]. Collectively, these results suggest that, in conjunction with CHOP and ATF5, ATF4 is also a critical mediator of the UPR^mt^ and may even act as the primary regulator of this response.

In tandem with this canonical signaling axis involving CHOP, ATF5, and ATF4, several non-canonical axes of the UPR^mt^ have also been described. The first axis, denoted as the antioxidant–UPR^mt^ axis, involves the mitochondrial deacetylase Sirtuin 3 (Sirt3) and the transcription factor FOXO3A [140,141,142]. Upon sensing the accumulation of matrix misfolded proteins, which subsequently leads to elevations in ROS production, Sirt3 becomes activated to deacetylate FOXO3A, triggering its nuclear localization and the upregulation of antioxidant enzymes, including superoxidase 2 (SOD2) and catalase [140,141,142]. As mitochondria contain different compartments, misfolded proteins within the intermembrane space will trigger a compartmental-specific axis, mediated by ERα [143]. This axis is triggered by the accumulation of misfolded proteins in the mitochondrial intermembrane space (IMS), leading to ligand-independent activation of ERα via phosphorylation at Serine 167 [143]. Activated ERα localizes to the nucleus to induce the expression of IMS proteases, such as high-temperature requirement A2 (HtrA2) [143]. An increase in ROS production within the IMS also triggers an ERα-specific response through AKT signaling to enhance the expression of Nuclear Respiratory Factor 1 (NRF1) and related antioxidant proteins [143]. Collectively, these UPR^mt^ pathways function in tandem to mitigate stress and preserve organellar integrity.

### 4.6. ISR and UPR^mt^ Interconnection

Considerable evidence from mammalian cells underscores the central role of ISR in coordinating the UPR^mt^ during mitochondrial stress, as mentioned previously. This interconnection is largely mediated by the mitochondrial stress-induced phosphorylation of eIF2*α*, which enables the preferential induction of master regulators CHOP, ATF5 and ATF4, coordinating a downstream UPR^mt^-specific response [44,98,100,144,145]. Collectively, these findings make it clear that the UPR^mt^ in mammals relies on the ISR-dependent enhancement of the master regulators. Notably, in *C. elegans*, ISR activation appears to be largely dispensable for UPR^mt^ induction [146], as manipulation of the eIF2*α* kinases during various mitochondrial perturbations does not abolish the response, suggesting a species-specific divergence for ISR requirement [70].

Beyond this regulatory paradigm, evidence in several cell types during general stress suggests that the expression and activation of each transcription factor are not independent of each other, forming a complex regulatory network [98,100,147]. For example, ATF4 deficiency blunts the stress-responsive induction of ATF5 and CHOP [52,98,100,144,147], while ATF5 expression requires both ATF4 and CHOP [98,100,144,147]. Prolonged stress, however, can trigger a CHOP-mediated negative regulation of ATF4 via direct heterodimerization through their bZIP domains, forming a negative feedback loop. This interaction has been shown to attenuate ATF4-dependent activation of target genes involved in cellular homeostasis [98,148]. While this paradigm has been elucidated in the context of general cellular stress, during mitochondrial stress, however, this coordination is more divergent [93]. In the absence of ATF4, only the expression of ATF5 is blunted, with no effects on CHOP even during mitochondrial chaperone inhibition [93]. Similarly, this reduced expression of ATF5 is also observed in CHOP-deficient cells, thereby pointing to a coordinated regulation of ATF5 by ATF4 and CHOP [93]. Consistent with other cellular stressors, the absence of CHOP leads to elevations in the expression of only ATF4, confirming the role of CHOP as an ATF4-specific negative regulator [93]. Despite these insights, the precise coordination of such transcription factors under mitochondrial stress remains elusive. Resolving this interplay, alongside the specific contribution of each factor to the ISR/UPR^mt^ signaling and mitochondrial health, particularly in the context of skeletal muscle, is warranted.

## 5. The Mitochondrial Stress Response in Skeletal Muscle

Although the precise signaling pathways underpinning the ISR/UPR^mt^ have largely been explored in many cell and tissue types, comparative evidence in skeletal muscle is underwhelming. As skeletal muscle is a highly metabolically active tissue, it is particularly susceptible to changes in mitochondrial functioning, thereby necessitating the stress response as an essential component required to maintain skeletal muscle health [149,150]. Over the course of several years, work from both our laboratory and others has attempted to uncover the importance of the mitochondrial stress response in skeletal muscle. For example, early evidence from our laboratory showed that the ISR/UPR^mt^ in C2C12 myotubes can become activated in response to Tim23 gene manipulation, a protein responsible for coordinating protein import into mitochondria [151]. The elevation in ISR/UPR^mt^ signaling during protein import stress, as evidenced in aberrations in CHOP, ClpP and Cpn10 protein expression, reflects the requirement for the stress response program to become activated during disrupted import and thus mitochondrial stress [151]. This reduction in import capacity ultimately leads to a form of genomic imbalance, termed mito-nuclear imbalance, hindering the amount of nuclear-derived mitochondrial proteins that can be incorporated into the mitochondria, thus impeding ETC supercomplex formation and functioning [152]. These findings were among the first to emphasize the importance of the mitohormetic stress response program in responding to defects in mitochondrial functioning, particularly in skeletal muscle [151]. In a similar oversimplified context, during various instances of mitochondrial stress, either mediated through pharmacological or genetic manipulation, the activation of the ISR/UPR^mt^ in skeletal muscle is necessary to mitigate detrimental effects to the mitochondrial reticulum, thereby preserving muscle health [29,153,154,155,156,157,158,159,160,161,162,163].

In the context of ISR/UPR^mt^ regulators, the previous literature from our laboratory also demonstrated a critical requirement for these factors to be present in skeletal muscle under basal, unstressed conditions, thereby solidifying their role as pertinent regulators of both mitochondrial and skeletal muscle health. For example, in C2C12 myotubes, the absence of ATF4 led to severe reductions in mitochondrial respiration, elevations in ROS emission, as well as overall incapacity to initiate mitochondrial quality control regulation [164]. Similarly, rodent muscle devoid of ATF5 experienced reduced mitochondrial OXPHOS capacity, an inability to regulate ROS production, and a suppression of antioxidant enzyme expression, alongside the blunted capacity to initiate and respond to common transient mitochondrial stressors (i.e., contractile activity and acute muscle disuse) [165,166,167]. Although these factors are deemed necessary in the maintenance of mitochondrial and muscle health, the precise signaling cascade leading to their activation, outside of their translational regulation, is still largely uncharacterized.

While the activation of the ISR/UPR^mt^ in response to mitochondrial stress is seemingly critical in the maintenance of muscle health, the sustained or prolonged activation of these stress responses can induce a detrimental cellular program and promote skeletal muscle atrophy. This consequence is dependent on the duration and nature of the stimulus, as an aspect of maladaptive hormesis, consummating a catabolic environment for muscle. The shift in the cellular outcome is largely determined by the ISR/UPR^mt^ transcriptional regulators, ATF4 and CHOP; their activity; and related subsequent dimer partners, amongst a plethora of unknown components. While these parameters are hypothesized to be the major regulators and determinants of the catabolic program in muscle, more evidence is required to solidify these postulations and identify the precise switch governing the shift in cellular outcome. Understanding the dichotomous nature of the ISR/UPR^mt^ program is necessary in order to fully appreciate the importance of this stress response program in maintaining mitochondrial health during contractile activity, muscle disuse, and aging. In the next sections of this review, we will highlight how the ISR/UPR^mt^ program can become mechanistically activated during these challenged states and the outcome of the program in relation to muscle health.

### 5.1. Activation of the ISR/UPR^mt^ During Acute Exercise

Acute bouts of exercise create a robust state of mitochondrial stress in skeletal muscle [168]. During this period, the energetic demand of contracting fibers transiently exceeds the capacity of the mitochondrial network, thereby engaging mitochondrial quality control pathways, including stress-responsive signaling mechanisms [169]. Indeed, evidence in skeletal muscle has exemplified that the ISR/UPR^mt^ can become activated in response to acute exercise-induced stress to induce a mitohormetic response. In C2C12 myotubes, a single bout of acute contractile activity leads to the upstream initiation of the ISR/UPR^mt^, as evidenced by the enhancement of associated transcription factors [164]. In a rodent model, Slavin and colleagues [165] determined that in response to an acute exhaustive bout of treadmill exercise, the ISR/UPR^mt^ was upregulated, primarily indicated by the nuclear localization of CHOP and ATF4. While the precise mechanisms governing activation of the ISR/UPR^mt^ axis are often attributed to elevated reactive oxygen species (ROS) and increased protein misfolding/mitonuclear imbalance (proteotoxicity), direct experimental evidence for the latter remains limited [170,171].

To date, the only robust and experimentally defined trigger of ISR/UPR^mt^ activation in skeletal muscle during acute exercise is a transient, pronounced increase in mitochondrial ROS emission. Acute exercise is well recognized as a potent mitochondrial stressor, and elevations in mitochondrial ROS during these bouts can reliably activate the ISR/UPR^mt^ pathway to stimulate a mitohormetic adaptive response [172,173]. Mechanistically, the elevation in mitochondrial ROS during contractile activity can be sensed by JNK2, which imparts the phosphorylation and thus activation of c-Jun to facilitate the activation of CHOP, a signaling axis previously discussed [174]. In the context of acute exercise, it has indeed been observed that elevations in JNK2 and c-Jun phosphorylation/activation, alongside concurrent increases in the mRNA expression/protein levels of CHOP, occur in rodent skeletal muscle following acute exercise [165,175,176]. Abolishment of JNK2 activation via an MAPK inhibitor, Sp600125, in rodent skeletal muscle reduces the overall responsiveness of the ISR/UPR^mt^ and related downstream targets following acute exercise, further fortifying this pathway involvement [177]. Concurrently, some evidence suggests that the ISR kinase, PKR, upon sensing mitochondrial ROS, can also stimulate the activation of JNK2 and further propagate this coordination [178]. Cumulatively, although mechanistically not distinguished, these findings indicate that the transient activation of ROS during acute exercise can, thereby, in proxy, stimulate mitochondrial stress response through JNK2 to therefore remediate mitochondrial homeostatic conditions.

In addition to direct kinase activation, recent evidence from our laboratory has indicated that acute exercise-induced mitochondrial stress can lead to the stabilization of ISR/UPR^mt^ regulatory factors, such as ATF4, in an attempt to promote the overall induction of this program [176]. Following acute hindlimb stimulation in rodents, stark elevations and the stabilization of ATF4 mRNA as a result of enhanced RNA-binding protein association with the stabilizing protein HuR were observed. This regulatory process was one of the first to demonstrate that acute contractile activity can not only elicit mitochondrial stress but also promote the activation of the ISR via the stabilization of ATF4 to facilitate mitochondrial and skeletal muscle adaptations during exercise.

Despite these findings, the precise mechanisms and distinct signaling cascades that govern hormetic activation of the ISR/UPR^mt^ following a single bout of contractile activity remain poorly defined. Nevertheless, the transient induction of these stress responses during acute exercise appears to restore mitochondrial homeostasis and prime the muscle for the beneficial adaptations that accrue with repeated/chronic exercise (i.e., training).

### 5.2. ISR/UPR^mt^ Mediated Adaptations Following Chronic Exercise

It is well recognized that exercise training induces beneficial adaptations within the mitochondrial reticulum and phenotypic changes in skeletal muscle, a phenomenon first described by John Holloszy in 1967 [179]. To date, the underlying mechanisms driving these adaptations are thought to converge on the activation of mitochondrial quality control processes, including mitochondrial stress responses. Indeed, early work from our laboratory was some of the first to show that chronically stimulated skeletal muscle possesses greater expression of ISR/UPR^mt^ downstream targets, mitochondrial chaperones, HSP60 and mtHSP70, indicating increases in mitochondrial folding capacity and thus responses to changes in the protein folding environment, an aspect of mitohormesis [180]. Following these foundational findings, in C2C12 myotubes, four days of chronic contractile activity (CCA) elicited increases in the expression of ISR/UPR^mt^ transcription factors CHOP and ATF4, alongside related downstream targets: mtHSP60, mtHSP70 and Cpn10 [181]. Likewise, in a rodent model of CCA, through time course evaluation, it was demonstrated that the expression of CHOP and ATF4 mRNA and protein levels was elevated between 1 and 7 days of the stimulus [175]. Interestingly, CHOP expression remained elevated throughout all 7 days, whereas ATF4 was reduced to control levels by day 5. Downstream targets—mtHSP70, Cpn10, HSP60, and ClpP—showed a consistent and sustained increase in mRNA levels throughout all 7 days of CCA [175]. These gradual shifts in ISR/UPR^mt^ activity over time reflect an ongoing need for this stress response program to drive mitochondrial hormesis and adaptations and thus improve the muscle phenotype, in parallel with other quality control pathways. Although the precise mechanisms by which ISR/UPR^mt^ confers benefits in skeletal muscle remain to be fully defined, current findings underscore the need to further investigate this stress response axis as a means to improve mitochondrial health during exercise.

## 6. The Intersection of Aging and the ISR/UPR^mt^

In contrast to the beneficial adaptations seen with exercise, the natural phenomenon of aging is often accompanied by a progressive reduction in both muscle mass and strength, termed sarcopenia [182]. Aging involves a gradual and cumulative decline in mitochondrial function marked by impairments in mitochondrial health, proteostatic machinery, and the uncontrollable generation of ROS [183,184]. With this being said, mounting evidence has demonstrated an increased basal activation of the ISR/UPR^mt^ pathway with age, which manifests as higher levels of phosphorylated eIF2*α*, as well as the upregulation of downstream effectors such as ATF4, CHOP and downstream ISR targets [185,186,187,188,189,190,191]. For instance, in both aged rodents and humans, there is an elevation in the basal expression of ATF4, and this activation is linked to prolonged mitochondrial stress, commonly attributed to the aging phenotype [187,188,192,193,194]. The recent literature has also emphasized similar trends with CHOP activation, alongside associated reductions in skeletal muscle health during aging, primarily in both the soleus and plantaris muscles of 18- and 24-month-old rodents [188,195]. This upregulation strongly correlates with reduced muscle mass, as evidenced by a negative correlation between CHOP expression and muscle weight. This highlights the link between chronically high levels of CHOP and muscle loss in aged tissue [188]. Interestingly, the UPR^mt^ machinery, including chaperones (HSP60/mtHSP70) and proteases (LONP1/YME1L1/CLPP), shows complex regulation during aging. Many reports indicate a decline in the expression of these protective proteins with age despite ongoing mitochondrial dysfunction [196]. However, some animal models display compensatory upregulation of UPR^mt^ genes alongside improved oxidative phosphorylation gene expression, suggesting heterogeneity in adaptive capacity [184,189].

During aging, the chronic activation of the ISR/UPR^mt^ should theoretically improve mitochondrial health, but it instead becomes immensely maladaptive, thereby promoting a hypercatabolic state. This association was first established by seminal work from Adams’ group, which highlighted that ATF4 can promote a pronounced skeletal muscle atrophy program and is the major ISR regulator capable of mediating muscle atrophy during aging [192,193,197,198]. While this relationship of ATF4 being a main driver of age-related muscle loss was recognized, the precise transcriptional dimer partner governing this program was not well defined until work by Ebert and colleagues [199] provided an elegant framework to assess this paradigm. Through various methodologies, ATF4 was shown to dimerize with multiple factors in skeletal muscle; however, the most prominent and critical factor required to facilitate the muscle atrophy program was C/EBP*β* [199]. This newfound ATF4-C/EBP*β* dimer pair was demonstrated to bind to conserved ATF-C/EBP composite response elements within the promoters of various atrogenes, including Gadd45α [199]. One of the most characterized axes downstream of ATF4 in skeletal muscle involves the transcriptional induction of Gadd45*α* (Growth arrest and DNA damage-inducible protein 45 alpha) [200,201,202]. The ATF4-C/EBP*β* dimer has been shown to directly bind to the proximal promoter of Gadd45*α*, driving robust increases in Gadd45*α* mRNA and protein in aged myofibers. Once upregulated, Gadd45α functions as a stress-sensing scaffold that activates the MAP kinase kinase MEKK4 (MAP3K4) [200,201]. In basal/young skeletal muscle, MEKK4 resides in an inactive conformation but becomes activated upon Gadd45α-binding during conditions of stress, such as during prolonged muscle inactivity or aging [200,201]. This protein–protein interaction generates a conformational change in MEKK4, which stimulates MEKK4 autophosphorylation at Threonine 1483, generating an active Gadd45a-MEKK4 kinase complex [200,201]. This activation ultimately leads to the sequential phosphorylation and activation of MKK3, MKK4, MKK6, and ultimately p38 MAPK, a potent catabolic signaling mediator in skeletal muscle [201,202,203,204]. While the upstream activation of this pathway is well characterized, the mechanism by which the downstream signaling cascade promotes muscle atrophy via biochemical mechanisms is not yet fully understood.

In addition to Gadd45α, ATF4 has been found to regulate the expression of p21 (Cdkn1a), a cyclin-dependent kinase inhibitor traditionally associated with cell cycle arrest [205]. Outside of its role in cell cycle regulation, in post-mitotic tissues, such as skeletal muscle, p21 expression is elevated, particularly during instances of disuse and aging and is purported to be a mediator of ATF4-driven related muscle atrophy [205,206]. This association was first elucidated by Ebert and colleagues, where AAV6-mediated overexpression of p21 in the tibialis anterior (TA) of young adult mice (3–4 months of age) was sufficient to recapitulate key features of sarcopenic muscle [207]. The precise mechanism governing this p21-mediated induction of muscle atrophy is still largely unknown; however, evidence has suggested that p21 can repress the mRNA-encoding spermine oxidase (Smox), which thereby reduces overall Smox protein levels [207,208,209]. Smox is a FAD-dependent oxidase enzyme required for regulating the conversion of polyamines and maintaining intracellular spermidine levels, a critical metabolite required for protein synthesis and cellular homeostasis [210,211,212]. In young, healthy muscle, Smox expression is elevated and required to promote muscle growth by influencing gene expression in a positive manner [207]. However, reductions in the expression of this enzyme ultimately lead to muscle fiber atrophy, potentially through the modulation of a pro-atrophy gene expression signature [207]. While the precise downstream signaling cascades governed by this pathway are not yet understood, it is clear that ATF4 is critical in propagating p21-mediated muscle atrophy with age.

CHOP expression, as previously mentioned, is largely induced during aging as a product of prolonged mitochondrial dysfunction [188]. However, while CHOP is a canonical regulator of apoptosis in other tissue models/types, and although some downstream targets of CHOP are elevated during aging in muscle, such as BAX, whether this program also contributes to the age-related muscle atrophy program is not well characterized [188]. Understanding how these stress response factors become elevated in aged muscle, their role in mediating muscle atrophy, and the precise signaling outcome remains a point to acknowledge and thus requires understanding the nature of the ISR/UPR^mt^ during conditions under prolonged activation.

## 7. The Activation of the ISR/UPR^mt^ During Muscle Inactivity

**Figure 3:**
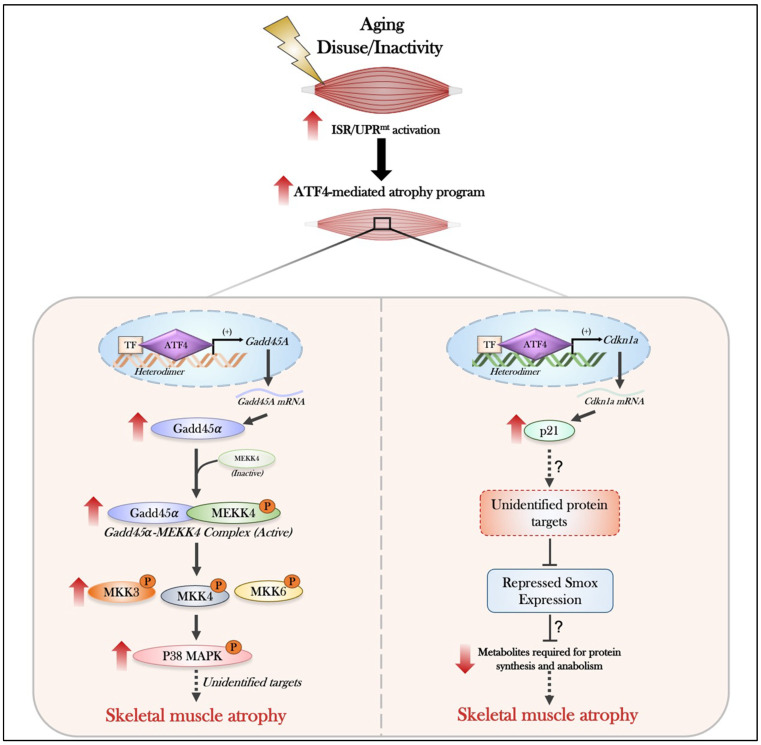
ATF4-mediated downstream mechanisms of skeletal muscle atrophy during aging and muscle disuse. Upon prolonged muscle disuse and aging, ATF4 mediates a transcriptional program to promote the expression of pro-atrophy genes, Gadd45α and p21. Gadd45α, when upregulated, leads to the activation of MEKK4 to form an active kinase complex, which is responsible for phosphorylating various MKK proteins (MKK3, MKK4 and MKK6) in skeletal muscle. Upon activation, these kinases will subsequently activate p38MAPK to induce skeletal muscle atrophy. Similarly, when p21 is upregulated, it can lead to the modulation of unidentified targets, which represses the expression of the Smox enzyme in an unknown manner, reducing metabolites required for protein synthesis and anabolism, consequently promoting skeletal muscle atrophy. Dashed arrows indicate unknown mechanisms/targets. Solid arrows indicate a known interaction/effect.

Similar to aging, chronic muscle inactivity, brought on by sedentarism, periods of immobilization, bed rest, or exposure to microgravity, promotes rapid muscle atrophy, alongside prominent declines in mitochondrial function and content [213]. The activation of the ISR/UPR^mt^ is also hyperactivated in skeletal muscle during periods of muscle disuse, and such regulation is demonstrated to further contribute to an atrophy program, findings emphasized in many studies [166,188,206,214,215,216,217,218]. For example, within the first 2–7 days of muscle disuse stimuli, such as hindlimb unloading or denervation, the activation of the ISR/UPR^mt^ is rapidly elevated, denoted by early changes in ATF4, CHOP, and related downstream targets [206,215,218,219,220]. This early upregulation is hypothesized to potentially reflect an adaptive attempt to maintain mitochondrial quality during low energy demand states, a typical consequence of sudden muscle disuse. Indeed, a recent study from our laboratory has emphasized the elevated expression of ISR/UPR^mt^-related proteins following 7 days of denervation [166]. However, beyond this early stage, the prolonged and continual upregulation of these factors shifts this adaptive phenotype into a catabolic program. This speculation was confirmed upon transcription factor deletion in muscle, whereby the absence of regulators during disuse stimuli prevents related atrophy [166,206,215,218,221]. While the mechanisms governing the specific ISR/UPR^mt^-induced atrophy program during disuse stimuli are still unknown, it is hypothesized that this program, driven in part by ATF4, is dependent on p21- and Gadd45α-mediated regulation [200,206,220,222]. With this in mind, understanding the precise mechanisms underlying the phenotypic switch and downstream consequences of the ISR/UPR^mt^ during disuse stimuli is still warranted (Figure 3). Future work should aim to discern this paradigm and focus on the signaling aspects governing this switch in skeletal muscle.

## 8. Modulation of the Mitochondrial Stress Responses to Improve Muscle Health

### 8.1. Direct Pharmacological Targeting of the ISR/UPR^mt^

Since the ISR/UPR^mt^ can be beneficial in specific cellular contexts, it is not surprising that the transient pharmacological activation of the ISR/UPR^mt^ improves mitochondrial stress resistance, owing to its hormetic nature. Small-molecule activators of GCN2 or HRI (e.g., halofuginone; nucleoside mimetics 0357 and 3610) in patient fibroblasts with MFN2 mutations augment ISR signaling to promote mitochondrial elongation and prevent pathological DRP1-mediated fragmentation, ultimately restoring network morphology [53]. In cardiomyocytes, acute pharmacological activation of eIF2*α* via Salubrinal reduces mitochondrial-complex-derived reactive oxygen species and confers robust protection against ischemia/reperfusion injury [223]. Within a rodent model, a small molecule, RDR03027, was shown to induce ISR activation in skeletal muscle, demonstrated by an upregulation of genes involved in amino acid biosynthesis and glutathione synthesis, alongside the myokine FGF21 [224]. These alterations led to enhanced energy consumption, prevented obesity, and therefore improved skeletal muscle architecture [224]. In contrast, with chronic stimuli, such as aging or prolonged muscle disuse, sustained ISR/UPR^mt^ activation becomes maladaptive, contributing to weakness and atrophy, as previously emphasized. In these settings, agents that blunt ISR-ATF4 overactivation can preserve muscle health. For example, in multiple models of muscle atrophy (fasting-, disuse-, and age-related), the natural compounds tomatidine and ursolic acid function as small-molecule inhibitors of ATF4-dependent pathways, reducing atrophy gene expression and improving muscle mass and function [192,225]. Although these strategies show strong efficacy in preclinical models, whether similar ISR-targeted or ATF4-modulating interventions will be safe and effective in humans remains an open question.

### 8.2. Exercise as Medicine

In addition to pharmacological interventions, exercise is seen as a potent physiological ISR/UPR^mt^ modulator, as previously mentioned, an aspect that becomes increasingly important during ISR/UPR^mt^ dysregulation. In particular, with aging, aerobic and high-intensity training induce the ISR/UPR^mt^ (elevating HSP60, LONP1, and YME1L1), which corresponds to improvements in mitochondrial metabolism, oxidative capacity, and the delay of sarcopenia-related muscle decrements [196,226]. These observations motivate the development of exercise mimetics (e.g., AMPK activators and mild mitochondrial stressors) that reproduce transient ISR/UPR^mt^ activation to enhance mitochondrial quality control in individuals who are incapable of undergoing exercise regimes, such as sedentary or frail individuals.

### 8.3. Nutritional Status and Amino Acids: Intersection with the ISR/UPR^mt^

Amino acids are indispensable anabolic signals, functioning as both building blocks for skeletal muscle and as direct regulators of the translational machinery. Despite this, the interplay between amino acid availability and the ISR/UPR^mt^ in regulating protein synthesis under conditions of compromised anabolic capacity, such as with aging, remains poorly understood. The ISR has been demonstrated to be highly sensitive to intracellular amino acid fluctuations. In MEFs, deprivation of essential amino acids, particularly leucine and arginine, activates the ISR kinase GCN2, thus enabling the activation of ATF4 [227]. This is accompanied by an upregulation of amino acid biosynthetic and transport genes, alongside transient mTORC1 suppression in an attempt to support stress-resolution [74,227]. During prolonged nutrient deprivation, the ISR becomes hyperactivated, leading to the enhancement of mTORC1 suppressors—REDD1 and Sestrin2—via ATF4, promoting the sustained inhibition of protein synthesis [74,228,229]. In contrast, amino acid supplementation can strongly stimulate/restore mTORC1 activity and activate ATF4 through a nutrient-driven, mTORC1-dependent mechanism [230]. Indeed, early findings highlighted that the promoter region of mTORC-related genes contains an abundance of ATF4-binding elements, suggesting that mTORC1 can activate ATF4 to sustain muscle growth [85]. This relationship was further confirmed during supplementation with leucine in myotubes, where the expression of ATF4 was drastically enhanced, coupled with an elevation in related amino-acid transport genes [230]. Nonetheless, these findings reveal a critical duality: ATF4 functions both as a sensor of amino acid insufficiency and as a downstream effector of mTORC1-driven anabolism. Whether amino acid supplementation can redirect ATF4 activity towards anabolism in compromised muscle (i.e., aged muscle) without inadvertently inducing catabolic targets remains unresolved. Future work should dissect ATF4’s transcriptional landscape across varying nutrient conditions in skeletal muscle to determine whether modulating the ISR/UPR^mt^ is a viable target for promoting muscle health.

## 9. Conclusions and Future Perspectives

**Figure 4:**
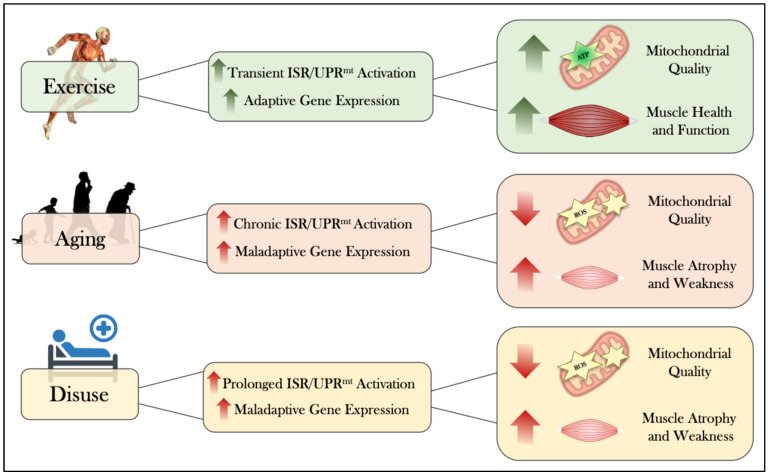
A summary of the metabolic events and physiological outcomes that occur with exercise, aging and disuse, with respect to ISR/UPR^mt^ activation. During exercise (acute and chronic), the ISR/UPR^mt^ is transiently activated, which propagates an adaptive gene program to promote enhancements in mitochondrial quality, adaptive capacity, and muscle health. Conversely, with aging and muscle disuse, chronic and prolonged activation of the ISR/UPR^mt^ is observed, culminating in a maladaptive program that does not support mitochondrial quality control and instead promotes the induction of muscle atrophy and weakness. Green arrows indicate a positive adaptation, red arrows indicate a maladaptation.

The ISR/UPR^mt^ is a central mitochondrial quality control pathway that regulates organellar health to preserve skeletal muscle function during physiological and pathological stress. Acute or moderate activation of these pathways, such as during exercise, enhances the capacity for mitochondrial adaptation, thereby supporting muscle performance. In contrast, chronic or excessive activation, as seen in aging muscle or during prolonged inactivity, can become maladaptive, driving atrophy, metabolic remodeling, and systemic disease progression (Figure 4). Although major advances have been made in understanding how the ISR/UPR^mt^ is modulated in mammalian cells, its specific role, regulation and downstream targets within skeletal muscle remain incompletely defined. Given the importance of mitochondrial health for the maintenance of skeletal muscle mass and function, several subjects warrant further investigation. Future work should delineate muscle-specific ISR/UPR^mt^ signaling nodes, including how different eIF2*α* kinases and transcription factors are engaged by distinct contractile or metabolic stressors. Translational studies should test whether fine-tuning ISR/UPR^mt^ activity, either enhancing transient activation or dampening chronic hyperactivation, can be leveraged therapeutically to preserve muscle mass and mitochondrial function in aging, inactivity, and muscle-wasting diseases. Together, the evidence reviewed underscores the ISR/UPR^mt^ as a central hub coordinating mitochondrial quality control and skeletal muscle adaptation across a spectrum of physiological and pathological conditions.

## Data Availability

No new data were created or analyzed in this study.

## Abbreviations

The following abbreviations are used in this manuscript:

5′UTR	5′ untranslated region
AARE	Amino acid response element
AIF	Apoptosis-inducing factor
AMPK	AMP-activated protein kinase
ATF4	Activating transcription factor 4
ATF5	Activating transcription factor 5
ATFS-1	Activating transcription factor associated with stress-1
BAK	Bcl-2 Homologous Antagonist/Killer
BAX	Bcl-2-associated protein X
BID	BH3-interacting-domain death agonist
BIM	Bcl-2 interacting mediator of cell death
c-Jun	Cellular jun
C/EBP	CCAAT-enhancer binding protein α/β/γ/δ
cAMP	cyclic AMP
CARE	C/EBP-ATF response element
CCA	Chronic contractile activity
ChIP-seq	Chromatin immunoprecipitation and sequencing
CHOP	C/EBP homologous protein
ClpP	Caseinolytic mitochondrial matrix peptidase proteolytic subunit
Cpn10	10 kDa chaperonin, mitochondrial
CRE	cAMP response element
CREB	cAMP response element-binding protein
CReP	Constitutive repressor of eIF2α cphosphorylation
DELE1	DAP3-binding cell death enhancer 1
DISC	Death-inducing signaling complex
DR4	Transmembrane death receptor 4
DR5	Transmembrane death receptor 5
DRP1	Dynamin-related protein 1
dsRNA	Double-stressed RNA
eIF2B	Eukaryotic translation initiation factor 2B subunit alpha
eIF2α	Eukaryotic translation-initiation factor-2 alpha
EndoG	Endonuclease G
ER	Endoplasmic reticulum
ERα	Estrogen receptor alpha
ETC	Electron transport chain
fCLIP-seq	Formaldehyde-mediated crosslinking and immunoprecipitation sequencing
FGF21	Fibroblast growth factor 21
FOXO3A	Forkhead box 3A
GADD34	Growth arrest and DNA damage-inducible protein 34
Gadd45α	Growth arrest and DNA damage-inducible protein 45 alpha
GCN2	General control nonderepressible 2 (serine/threonine protein kinase)
GDF15	Growth differentiation factor 15
GDP	Guanine diphosphate
GEF	Guanine exchange factor
GTTP	Gamitrinib-triphenylphosphonium
HRI	Heme-regulated inhibitor
HSP10	10 kDa heat shock protein
HSP60	60 kDa heat shock protein
HtrA2	High temperature requirement A2
HuR	Human antigen R
IMS	Intermembrane space
ISR	Integrated stress response
ISRIB	Integrated stress response inhibitor
JNK	c-jun N-terminal kinase
LONP1	Lon peptidase 1
MAP3K4	MAP kinase kinase MEKK4
MAPK	Mitogen-activated protein kinase
MEF	Mouse embryonic fibroblasts
MEKK4	Mitogen-activated protein kinase kinase kinase 4
Met-tRNA_i_	Methionyl-initiator tRNA
MFN2	Mitofusin 2
MKK3	Mitogen-activated protein kinase kinase 3
MKK4	Mitogen-activated protein kinase kinase 4
MKK6	Mitogen-activated protein kinase kinase 6
MOM	Mitochondrial outer membrane
MOMP	Mitochondrial outer membrane pore permeabilization
MQC	Mitochondrial quality control
mtDNA	Mitochondrial DNA
mtHSP60	60 kDa mitochondrial heat shock protein
mtHSP70	75 kDa mitochondrial heat shock protein
mTORC1	Mammalian/mechanistic target of rapamycin complex 1
mtRNA	Mitochondrial RNA
MURE	UPRmt response element
NADH	Nicotinamide adenine dinucleotide (reduced)
NRF-1/2	Nuclear respiratory factor-1 and -2
NuGEMPs	Nuclear genes encoding mitochondrial proteins
OAA	Oxaloacetate
OMA1	Optic atrophy 1
OXPHOS	Oxidative phosphorylation
p21	Cyclin-dependent kinase inhibitor 1 or CDK-interacting protein 1
PERK	Protein kinase R-like ER kinase
PIC	Preinitiation complex
PKR	Protein kinase RNA-activated
PNPase	Polynucleotide phosphorylase
PP1	Protein phosphatase 1
PP1c	Protein phosphatase 1 catalytic subunit
PUMA	p53 upregulated modulator of apoptosis
REDD1	Regulated in development and DNA damage responses 1
REs	Response elements
ROS	Reactive oxygen species
SIRT3	Sirtuin 3
Smox	Spermine oxidase
SOD2	Superoxidase 2
TOM	Translocase of the outer membrane
TRAIL	TNF-related apoptosis-inducing ligand
uORF	Upstream open reading frame
UPRmt	Mitochondrial unfolded protein response
YME1L1	YME1-like 1 ATPase
